# Organophosphate Pesticide Exposure and Neurobehavioral Performance in Agricultural and Nonagricultural Hispanic Workers

**DOI:** 10.1289/ehp.8182

**Published:** 2006-01-23

**Authors:** Joan Rothlein, Diane Rohlman, Michael Lasarev, Jackie Phillips, Juan Muniz, Linda McCauley

**Affiliations:** 1 Center for Research on Occupational and Environmental Toxicology, Oregon Health and Science University, Portland, Oregon, USA; 2 Oregon Child Development Coalition, Wilsonville, Oregon, USA; 3 University of Pennsylvania, Philadelphia, Pennsylvania, USA

**Keywords:** biomarkers, farmworkers, neurobehavior, occupational health, organophosphates, pesticides

## Abstract

Our understanding of the health risks of farmworkers exposed to pesticides in their work and home environments is rapidly increasing, although studies designed to examine the possible neurobehavioral effects of low-level chronic pesticide exposure are limited. We measured dialkyl phosphate urinary metabolite levels, collected environmental dust samples from a subset of homes, obtained information on work practices, and conducted neurobehavioral tests on a sample of farmworkers in Oregon. Significant correlations between urinary methyl metabolite levels and total methyl organophosphate (azinphos-methyl, phosmet, malathion) house dust levels were observed. We found the neurobehavioral performance of Hispanic immigrant farmworkers to be lower than that observed in a nonagricultural Hispanic immigrant population, and within the sample of agricultural workers there was a positive correlation between urinary organophosphate metabolite levels and poorer performance on some neurobehavioral tests. These findings add to an increasing body of evidence of the association between low levels of pesticide exposure and deficits in neurobehavioral performance.

In recent years, there has been increasing concern regarding the widespread use of pesticides in agricultural communities and potential impacts on public health. In the 1990s in the United States, some 2.5–5.0 million agricultural workers were exposed to organophosphate insecticides (Das et al. 2001). Scientific field investigations have focused on delineating the extent of exposure and potential health effects in agricultural and nonagricultural communities. Detectable levels of pesticides have been reported in home dust, primarily in families residing in agricultural areas (Bradman et al. 1997; McCauley et al. 2001; Quandt et al. 2004; Simcox et al. 1995). Bradman et al. (1997) found that diazinon and chlorpyrifos concentrations in house dust tended to be higher among farmworkers than among nonfarmworkers. Others have reported higher levels of pesticides in house dust in homes that are located closer to fields (Quandt et al. 2004) and in housing with larger numbers of farmworkers (Azaroff 1999; Lu et al. 2000; McCauley et al. 2001). After-work hygiene practices, such as leaving work boots outside and changing promptly from work clothes, have also been found to affect pesticide levels in the homes of farmworkers (McCauley et al. 2003).

Studies have also documented the presence of biologic markers of pesticide exposure in adults and children in agricultural communities (Arcury and Quandt 2003; Azaroff 1999; Loewenherz et al. 1997; O’Rourke et al. 2000) and differences among levels of exposure in residents of agriculture and nonagricultural communities. Although the association between acute exposure to pesticides and neurotoxic effects is well known (Lotti 2000), the potential effects of chronic low-level exposure are less well established (Alavanja et al. 2004).

Neurobehavioral (NB) test batteries have frequently been used to examine NB effects of acute pesticide exposure in adult working populations. Individuals with histories of toxic exposures to organophosphates have shown a consistent pattern of deficits on measures of motor speed and coordination, sustained attention, and information processing speed (Reidy et al. 1992; Rosenstock et al. 1991; Savage et al. 1988; Steenland et al. 1994; Wesseling et al. 2002). Fewer studies have examined the effect of long-term, low-level exposure to pesticides on nervous system functioning, but NB changes have been reported in sheep farmers (Stephens et al. 1995), greenhouse workers (Bazylewicz-Walczak et al. 1999), tree fruit workers (Fiedler et al. 1997), and farmworkers in Florida (Kamel et al. 2003). These studies have found deficits in measures of sustained attention, information processing, and motor speed and coordination. An examination of a group of cotton pesticides applicators in Egypt presumed to have high exposures, found a broad range of deficits, including visual motor speed, verbal abstraction, attention, and memory (Farahat et al. 2003).

Although these studies represent increasing knowledge regarding the association between pesticide exposure and neurologic health end points, few studies have reported the association between environmental exposures, biomarkers of exposure, and neurologic performance. We conducted an investigation of migrant farmworkers in Oregon and included measures of environmental exposure, biomarkers of exposure, and NB performance.

In this study, we hypothesized that *a*) significant correlations would be found between the amount of organophosphate residues in house dust and the levels of organophosphate metabolites in urine of adult farmworkers living in an agricultural community; *b*) the NB performance of Hispanic immigrant farmworkers exposed to organophosphates would be lower than that observed in a nonagricultural Hispanic immigrant population when controlled for demographic factors such as age and education; and *c*) within the agricultural workers, there would be a positive correlation between urinary organophosphate metabolite levels and poorer NB performance.

## Materials and Methods

### Target communities.

The agricultural community at Hood River is a productive and long-established agricultural community primarily producing pears and apples and located along the Columbia Gorge, approximately 100 km east of Portland, Oregon. The farmworker population in Hood River tends to consist of newly arrived and more permanent Hispanic residents who live in cabins, trailers, single-and multifamily homes, or apartments that are located in or alongside orchards. Harvesting of tree fruit begins in August and extends through October. The study was conducted as a partnership with the Oregon Child Development Coalition, which is the grantee for the Oregon Migrant Head Start Program. Ninety-six farmworkers were recruited by community members of the Migrant Head Start program in Hood River. All attendees at parent meetings at Migrant Head Start who had a child enrolled in a Migrant Head Start program and were currently working in the orchards, fields, and nurseries were invited to participate. Participants ranged from 20 to 52 years of age and were all originally from Mexico. Some of the parents first arrived in the United States in 1970, and some parents had just arrived for the first time in 1998. After the families were recruited, they were scheduled for a home visit, at which time questionnaires were administered and dust samples collected.

To compare performance on NB tests, we recruited immigrant workers from Newport, a tourist coastal town with little agriculture in Lincoln County, Oregon. The Hispanic workforce in Lincoln County consists of immigrant workers who are employed primarily by the local hotels and tourist industry. Most of these individuals came to Oregon 6–8 years earlier after they were solicited in Mexico to work in the Oregon fish canning industry. When the canning business declined, these workers remained in Newport to work in hotels and restaurants. They were recruited for this study by a community member with the support and partnership of the Hispanic community organizations Centro de Ayuda and Un Paso Adelante. The individuals were recruited one on one through word of mouth, community contacts, and neighborhood grocery stores. Workers were eligible to participate in the study if they had not worked in agriculture during the previous 3 months (including nurseries, farms, and fruit packing plants), were 18–50 years of age, had not attended school in the United States other than English-as-second-language classes, did not use a computer at work, and had never had an acute illness associated with pesticide exposure.

All biologic samples and NB assessments of both farmworkers from Hood River and the control group from Newport were conducted in the evenings after their workday. Participants were paid an incentive for participating in this study. The study protocol and procedures for informed consent were reviewed and approved by the Oregon Health and Science University Institutional Review Board (protocol 4216) and complied with all applicable requirements of the U.S. regulations.

### Data collection.

Spot urine samples for pesticide metabolite analysis were collected from farmworkers once during the summer and again in the fall. Samples were collected from each farmworker at the Migrant Head Start center in the evening after work just before taking the NB tests. Samples were labeled, and transferred on ice to the Oregon Health and Science University analytical laboratory. Urine specimens were adjusted to pH 3.0, aliquoted into test tubes, and stored at −20°C until extraction and analysis.

House dust samples were collected from a subsample of 26 farmworkers’ homes during the same week as collection of the first urine sample. Azinphos-methyl [AZM; trade name Guthion; Chemical Abstracts Service (CAS) No. 86-50-0], chlorpyrifos (CAS No. 2921-88-2), and phosmet (trade name Imidan; CAS No. 732-11-6) are used to control orchard pests such as coddling moth and are applied two to four times from May through August in the Hood River community. We timed our collection of home dust samples and urine samples to coincide with the middle of the growing season and the time that pesticide spraying applications were being applied to crops in the Hood River community. Dust samples were collected using a high-volume, small surface sampler (HVS3) as described in Lewis et al. (1994) and Simcox et al. (1995). All samples were collected from carpeted areas in the most commonly used play area for their children and living area for adults. All samples were collected in Teflon bottles (E.I. Dupont Company, Wilmington, DE) by vacuuming a measured area on a rug or carpet designed to collect an approximate 5-g sample. Samples were transported to the lab in a refrigerated cooler and stored below −20°C before analysis.

Both the farmworkers and control participants received NB testing in the evenings after work. Controls were tested once in spring and the farmworker participants were tested twice, in the summer and fall. Although our farmworker and control populations were recruited from two different communities, we assembled similar NB testing environments in both the Newport and Hood River testing sites. Testing stations were set up by using panel dividers to partition tables into different stations. Each station contained a computer, response unit, and headphones. Instructions on how to complete the computerized tests were given in Spanish. Four to six participants were tested at one time in air-conditioned meeting rooms. NB tests were selected from the Behavioral Assessment and Research System (BARS). BARS is a computerized test system that employs both written and spoken instructions (both via computer) (Rohlman et al. 2003). To minimize the adverse impact of working on an unfamiliar device such as a computer keyboard, a durable response unit with nine buttons is placed over the keyboard (pictured in Anger et al. 1996). The BARS test instructions have been translated into Spanish, recorded, and digitized. Instructions were written in Spanish on the screen and also delivered simultaneously through headphones. The eight BARS tests include measures of psychomotor functioning (finger tapping, simple reaction time, and progressive ratio) and measures of cognitive functioning (symbol-digit, digit span, selective attention, serial digit learning, and continuous performance).

### Laboratory analysis.

Dust samples were put through a sieve, extracted with organic solvents, cleaned up using gel permeation chromatography, and analyzed on a Hewlett-Packard (Palo Alto, CA) model 5890 gas chromatograph equipped with a pulse flame photometric detector (OI Analytical, College Station, TX). The organophosphates AZM, diazinon, chlorpyrifos, malathion, methyl parathion, and phosmet were confirmed with gas chromatography (GC)/mass spectrometry mass-selective detector in single ion monitoring mode. Specific methods for sample extraction and sample cleanup, involving filtration and gel permeation chromatography column cleanup and GC analysis, have been previously described (Moate et al. 2002). The limits of detection (LODs) for the six organophosphates were 0.01 μg/gm for diazinon, malathion, chlorpyrifos, and methyl parathion and 0.10 μg/gm for AZM and phosmet.

Urine was analyzed for five dialkyl phosphate (DAP) metabolites: dimethylphosphate (DMP), diethylphosphate (DEP), dimethylthiophosphate (DMTP), diethylthiophosphate (DETP), and dimethyldithiophosphate (DMDTP). Urine samples were prepared for GC analysis according to a modified method of Moate et al. (1999). Aliquots of the samples underwent azeotropic distillation with methanol and evaporation under a nitrogen stream. Sample extracts were then derivatized with 2,3,4,5,6-pentafluorobenzylbromide to convert phosphate acids to esters. Extracted samples were analyzed on a gas chromatograph (Hewlett-Packard model 5890) equipped with a pulsed-flame photometric detector (OI Analytical). The LOD for each of the metabolites was calculated from the instrument response factor corresponding to a concentration having a peak area three times the baseline noise (blank signal). The LODs for the five metabolites were 4.0 ng/mL (0.032 μmol/L) DMP, 2.0 ng/mL (0.013 μmol/L) DEP, 2.2 ng/mL (0.015 μmol/L) DMTP, 1.6 ng/mL (0.010 μmol/L) DMDTP, and 1.6 ng/mL (0.0095 μmol/L) DETP. The average extraction efficiencies of the five metabolites were, respectively, 45, 84, 97, 96, and 93%. Urine samples were also analyzed for creatinine concentrations (milligrams per deciliter), which were determined by the modified Jaffe reaction creatinine procedure No. 555 (Sigma Chemical Company, St Louis, MO).

### Quality control/quality assurance.

Quality control data generated for each set of urine samples provided an overall assessment of precision, accuracy, and reliability of the method. We conducted spike sample recoveries and urine blank analysis for every set of 12 samples. Urine samples known to contain low levels of DAP were used for blanks and for spike recoveries. Urine samples were spiked with DAP reference standards varying in concentration from 2 to 50 ng/mL.

### Data analysis.

We examined the distribution of creatinine and excluded urine samples less than the 5th percentile (26.45 mg/dL) or greater than the 95th percentile (235.5 mg/dL) from further analysis because of concerns of hydration state and metabolic disorders (Loewenherz et al. 1997; Lu et al. 2001). The primary organophosphates applied during the spring and summer season in the agricultural regions under study were AZM and phosmet, both of which break down into the methyl DAP metabolites (DMTP and DMDTP). Therefore, for urine samples, molar equivalent concentrations of the DMTP and DMDTP metabolites were summed to create a measure of thiomethyl DAP concentration. Nondetectable levels of urinary metabolites were replaced by one-half the appropriate LOD before taking the sum.

For house dust samples, residues associated with AZM, phosmet, and malathion (the most common agricultural organophosphates used in the study region) were added together to form a summary measure of pesticides in the house dust. Each of these pesticides metabolizes into the thiomethyl DAPs. Nondetectable levels of dust residues were replaced by one-half the appropriate LOD before taking the sum.

The association between methyl phosphates in house dust and thiomethyl concentrations in urine was evaluated using Spearman’s correlation. The difference in urinary thiomethyl metabolites between the first sampling period [summer, time 1 (T1)] and the second sampling period [fall, time 2 (T2)] was evaluated with a Wilcoxon signed-rank test. This test suggested that thiomethyl metabolites from T1 and T2 could be combined for subsequent analyses. Subjects with valid creatinine levels from both sampling periods had their metabolite levels averaged over the two samples; subjects with a valid creatinine level from only one sample contributed metabolite data from only that sample. The partial correlation (Rao 1973) was computed to examine the association between NB test performance from T1 and the averaged thiomethyl metabolite levels after accounting for the effects of sex, age, and education in the subject’s country of origin (age and education were treated as continuous variables; sex was a two-level factor). This analysis was conducted for subjects having a valid creatinine level during at least one of the two sampling periods. Differences on each NB test between agricultural (AG) and nonagricultural (non-AG) groups were assessed using multiple linear regression models involving age, sex, years of education in the subject’s country of origin, and AG versus non-AG status. Three interactions between AG status and each of the other predictors were also included in the initial model and simultaneously tested for significance using a partial *F*-test (Netter et al. 1989). If the test was significant (*p* < 0.10), then each interaction was separately examined and retained in the model if individually significant (again, at the *p* < 0.10 level). Adjusted values reported from the regression model reflect the mean score on each NB test for a 25-year-old subject with 6 years of education in his or her country of origin.

To increase the power to detect effects of exposure between the AG and non-AG population, we derived a summary index of overall NB performance from 11 of the 16 NB test items (digit span forward, digit span reverse, progressive ratio, reaction time, selective attention interstimulus interval, serial digit learning, symbol-digit, preferred-hand finger tapping, nonpreferred-hand finger tapping, alternating-hand finger tapping, and continuous performance percent hits). The items for the summary index were chosen to provide an equal representation of all the multiple measures in the test battery and were chosen before identification of the individual items that were statistically different between the two comparison groups. Measurements for each test were first standardized by subtracting the mean and dividing the difference by the sample SD. Tests involving latency measures had the signs of the standardized measurements reversed to provide consistency with the other measures (higher numbers indicating better performance; lower numbers, weaker performance). We computed the summary index as each subject’s average standardized score from the test items divided by the SE. The summary index was similarly analyzed to determine whether significant partial correlations existed with thiomethyl metabolites or if significant differences existed between the AG and non-AG groups.

All *p*-values are two sided unless otherwise indicated. One-sided *p*-values were used in cases where the means or correlations were anticipated to follow a prechosen trend. All analyses were performed with R (version 1.9.1; R Development Core Team 2004).

## Results

Ninety-nine farmworkers attended the parent meeting at Head Start and were approached for study participation, with only three declining to participate. Fifty-five controls were recruited for the study, but 10 were excluded because they were working in landscaping or tree planting (forestry), had no formal education in Mexico or the United States, or were not available during scheduled testing times. All farmworkers were immigrants from Mexico, and the controls were primarily from Mexico (two participants were from Guatemala and one unknown). There was no significant difference in the ages of the two groups: farmworkers, 20–52 years of age (mean ± SD = 29.7 ± 6.89); controls, 19–48 years of age (mean ± SD = 27.8 ± 6.19). The control group averaged 1.1 years more education than the farmworkers (*p* = 0.04; 95% confidence interval, 0.026–2.2 years more). The percentage of males in the two groups was not significantly different (*p* = 0.33). The mean time since first arrival in the United States was 9.8 years for farmworkers and 7.3 years for controls.

### Pesticide residue in house dust.

Our pesticide data included carpet dust samples from 26 farmworkers’ homes. Data on the six organophosphates we analyzed are reported in Table 1. At least one of the six organophosphates was detected in each of the homes. Phosmet, with a median detected concentration of 4.40 μg/g, was detected in 25 of the 26 homes (96%). AZM was detected in 18 of the 26 homes (69%) but had a higher detected median concentration (5.30 μg/g). Neither of these organophosphates is registered for residential use, and spray records from local growers in the area reported orchard application of phosmet and AZM two to four times from May through August. The organophosphates chlorpyrifos, parathion, malathion, and diazinon were detected at frequencies between 62 and 92% but at median detectable concentrations several times lower than found for AZM or phosmet.

### Urinary metabolite levels.

The two sampling periods with farmworkers provided a total of 172 urine samples (93 samples at T1, 79 samples at T2). We analyzed the urinary metabolites for all samples, but two samples were of insufficient volume for subsequent creatinine analysis. The distribution of creatinine levels in the remaining 170 samples was examined, and urine samples less than the 5th percentile (26.45 mg/dL) or greater than the 95th percentile (235.5 mg/dL) were excluded from further analysis because of concern about hydration state and metabolic disorders. This restriction reduced to the number of valid urine samples to 84 and 68, respectively, for T1 and T2; 88 subjects had valid urine samples for at least one of the two sampling periods.

DMTP was the most commonly quantified organophosphate metabolite (Table 2). The percentage of the complete sample above the LOD was 97 and 100%, respectively, for T1 and T2. When computed for samples with valid creatinine levels, the median concentration of the combined thiomethyl metabolites (DMTP, DMDTP) was 0.43 μmol/L at T1 (*n* = 84) and 0.48 μmol/L at T2 (*n* = 68); the median increased to 0.56 μmol/L when data from the two time periods were averaged together and the sample was broadened to include subjects with at least one valid creatinine measurement (from T1 or T2).

No significant differences were found between the median concentrations of thiomethyl metabolites from the two periods (*p* > 0.20 for both DMTP and DMDTP, Wilcoxon signed-rank test). Males tended to have higher levels of DMTP and combined thiomethyl metabolites at both time points compared with female farmworkers.

### Correlation of home dust samples and urinary metabolite levels.

**Figure 1:**
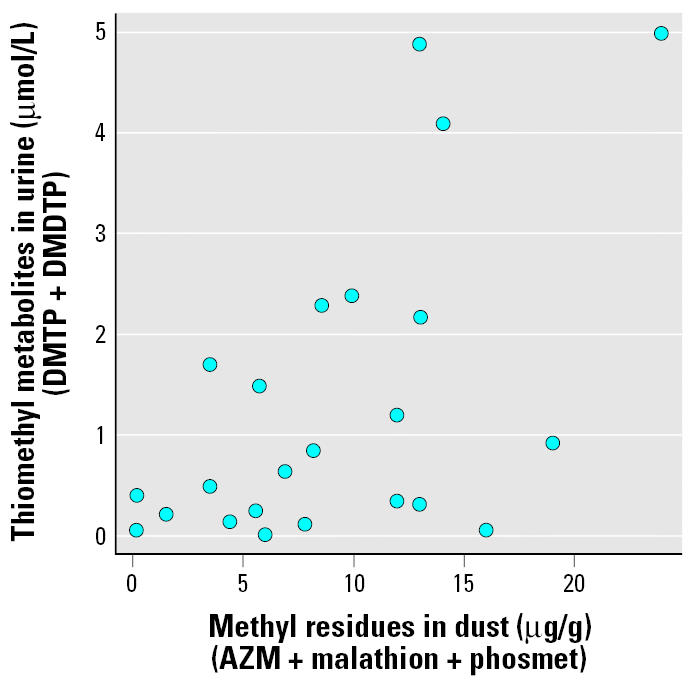
Scatter plot of combined methyl residues found in dust versus thiomethyl metabolite concentration in urine (*n* = 23 pairs). Spearman’s correlation is 0.47 (one-sided *p* = 0.013).

Twenty-three of the 26 carpet dust samples had pesticide residues that could be paired with the combined molar concentration of thiomethyl metabolites (DMTP and DMDTP) from valid urine samples. A moderate but significant positive correlation existed between these 23 pairs of methyl pesticides (sum of AZM, phosmet, and malathion) and their metabolites (micromoles per liter) (Figure 1). The impact of the three uppermost points observed in Figure 1 is reduced when summarized using Spearman’s correlation (*r_s_* = 0.47, one-sided *p* = 0.013).

### Correlation between NB performance and urinary metabolite levels.

Ninety-two farmworkers (51% male) and 45 controls (60% male) completed NB tests. NB performance was compared with the combined thiomethyl metabolites (DMTP + DMDTP) averaged across the two urine samples. After adjusting for age, sex, and years of education, poorer performance on five NB tests was associated with higher levels of the average combined thiomethyl metabolites: selective attention latency, symbol-digit latency, preferred-hand finger tapping, alternating-hand finger tapping, and continuous performance hit latency (Table 3).

### Comparison of NB performance between farmworkers and controls.

Overall, non-AG controls performed better on 12 out of 16 NB measurements compared with 92 farmworkers (Table 4). Multiple linear regression was used to compare performance on the NB tests between the AG and non-AG groups while controlling for age, years of education in country of origin, and sex. Interactions between these three covariates and employment in agriculture may have also been included if significant (*p* < 0.10).

Significant interactions between agricultural status and the covariates were found on the serial digit learning test [AG × age: *F*(1,122) = 3.96, *p* = 0.049; AG × sex; *F*(1,122) = 4.28, *p* = 0.041], the symbol-digit test [AG × education: *F*(1,127) = 4.20, *p* = 0.043], and preferred-hand finger tapping [AG × sex: *F*(1,129) = 4.73, *p* = 0.031]. Table 4 contains scores for AG and non-AG groups adjusted to reflect the mean response for a 25-year-old individual with 6 years of education; results are shown separately for each sex in cases where a significant AG × sex interaction was found. Interactions involving agricultural status and either age or education are shown in Table 5. Scores on the symbol-digit (latency) tests improved (i.e., decrease) significantly with increasing education for both groups, but the AG group showed greater benefit from each additional year of education. On the serial digit learning test, the two groups have linear trends that diverge with respect to age, although neither trend is significant. The summary index, derived from 11 of the 16 NB tests, also exhibited an AG × sex interaction [*F*(1,129) = 6.51, *p* = 0.012].

## Discussion

To the best of our knowledge, this is the first study to report a correlation between occupation, pesticide residues in house dust, biologic indicators of exposure, and effects on NB performance. Although the sample is limited to a migrant farmworker population in Oregon, the results link multiple points on the exposure–health effects pathway that underlies studies of environmental and occupational exposure and health. We have previously reported that the pesticide residues in the house dust of farmworker homes in Hood River exceed those found in homes in other agricultural and nonagricultural regions of Oregon (McCauley et al. 2001). Farmworkers are exposed to pesticides from both work practices and living in housing close to agricultural fields. Although not measured in the present study, we have previously reported that the average distance of farmworker housing to agricultural fields is 15 m in the Hood River community (McCauley et al. 2001).

Application dates in the spray records from orchards in the Hood River neighborhoods surrounding the homes of the participants in this study indicate that applications of phosmet and AZM in this region occurred within 1 week of our dust sample collection. The variability of levels of pesticide in household dust according to season and spraying activity has not been well established and would be difficult to ascertain in most agricultural communities because field-specific information on product name, amount applied at each location, and the crop type are not available. At this time, only six states have legislation requiring extensive reporting of pesticide use, including individual grower use.

The correlation found between environmental contamination and levels of urinary metabolites is a further example of a take-home pathway of pesticide exposure and points to the importance of home hygiene practices to decrease take-home exposures (Coronado et al. 2004; McCauley et al. 2003). Overall, the correlation found is impressive given that current pesticide levels in house dust are merely a marker of exposure history and not a direct measure. We have previously reported in a small sample of growers in Hood River a significant correlation between self-reported hygiene practices and levels of pesticides in home dust (McCauley et al. 2003). It is important that health education messages to this community include information on measures that growers and farmworkers can take to prevent home contamination (Coronado et al. 2004; McCauley et al. 2003; Thompson et al. 2003). Of particular importance is the removal of work shoes outside of living areas, changing from work clothes and showering upon arriving home, frequent mopping of hard floor surfaces, and steam cleaning carpets when appropriate. This was a community-based participatory research study, and all the study results have been shared with advisory board members and farmworkers in the community. We also have reported on the development and dissemination of a training video that emphasizes take-home pesticide contamination and the importance of home hygiene practices (Napolitano 2002).

Among individuals of similar age and education, we found that nonagricultural adults performed better on most of the NB measures that we included in our testing protocol. Measuring NB performance in immigrant, non-English-speaking populations and obtaining comparable comparison groups are always scientific challenges. Participants from both groups in this study had been residing in the United States for comparable periods of time and had similar years of education. Both groups emigrated from similar areas of Mexico. Both groups tend to maintain strong ties with the recently immigrated families within their community. Most important, the Latino community organizations within the state informed the researchers on the similarities of these two groups and how the tourism workers would be an appropriate comparison population to the farmworkers. Both groups are very similar in their engagement in low-paying jobs such as agricultural work, housekeeping or janitorial services for the hotel industry, or restaurant workers in a tourism community.

These findings add support to a growing body of evidence of NB changes in occupational groups chronically exposed to pesticides (Bazylewicz-Walczak et al. 1999; Fiedler et al. 1997; Kamel et al. 2003; Stephens et al. 1995). A pattern of poorer performance among farmworkers was observed on most measures in our test battery. The performance measures that we found to be associated with agricultural work are also measures that have been shown to be associated with low-level, chronic exposures to pesticides, including sustained attention, information processing, and motor speed and coordination.

In research conducted to date, measuring differences in performance on highly specific NB tests has been the most common methodologic approach. The correlation between the types of deficits seen, replication of specific deficits across studies, correlation with animal models, and the toxicologic effects of these chemicals is no doubt of extreme importance. However, Alavanja et al. (2004) and Heyer et al. (1996) point out the utility of grouping results of NB tests as a tool in interpreting findings because it will increase the power to detect effects of exposure in epidemiologic investigations. We found the summary index useful in discerning differences in exposure groups and sex effects. We did, however, construct the summary index *a priori* to reflect components of all the major areas being tested. Selective attention latency and continuous performance latency were not part of the summary index but showed a significant correlation with the levels of urinary metabolites. Future methodologic investigations of the utility of a NB summary index are needed.

Interactions have been found between NB performance and demographic variables such as age, education, and sex (Anger et al. 1997). In the present study, the NB summary index score was significantly affected by the sex of the farmworker. The reasons for this effect are unclear. Previous studies of NB performance in farmworkers have generally assumed that observed deficits are a result of pesticide exposure (Kamel et al. 2003), and significant sex effects in humans have not been reported. Several studies of organophosphate exposure in rats have demonstrated differential effects of sex (Dam et al. 2000; Levin et al. 2001, 2002). In this study, male farmworkers tended to have higher levels of methyl metabolites than female workers. These differences could be contributed to cultural, exposure, metabolic, or other yet unidentified factors. It is also important to consider genetic differences in the ability to metabolize organophosphate pesticides (Furlong 2000). Future studies will examine polymorphic differences and their relation to factors in the exposure pathway.

The design of this study has several limitations. Pesticide-specific information cannot be derived from quantitatively measuring the total urinary DAP metabolite levels, and because individual pesticides differ in toxicity, these cumulative measurements cannot be viewed as a measure of total toxicity (Wessels 2003). Furthermore, these biomarkers reflect recent exposure via all pathways over a very short time frame. Pesticide regulation and use are changing, and the pesticides found in home dust will vary according to the types of crops grown in an area. Therefore, similar results may not be found in all agricultural communities. For example, after this study, AZM became less frequently used, and the pattern of pesticides that we found in home dust in the same communities changed. If possible, future studies should include markers of specific organophosphate pesticides rather than DAPs. Reporting systems need improvement so that occupational spray records can be correlated with urinary levels of pesticides.

Urinary metabolites of organophosphate pesticides have a relatively short half-life, and it is unlikely that the performance on the NB tests at a given test session is a temporary influence on performance measured at that given point in time. Rather, the urinary metabolite levels should be considered a marker or approximation of a level of exposure, just as the NB measures are a marker of performance that could change from one testing session to another. So although one could suggest that the differences observed between agricultural and nonagricultural communities is due to pesticide exposure, additional studies are merited.

## Conclusions

This study links multiple points on the pesticide exposure–health effects pathway that underlies studies of environmental and occupational exposure and health. Although there have been increasing reports in the literature of the extent of pesticide exposure in agricultural communities, few studies have included markers of potential health effects. The correlation between levels of pesticides in the home and pesticide urinary metabolites points to significant prevention and education implications, and these messages are important to the farm-worker and other agricultural communities. To our knowledge, this study is the first to report a significant correlation between low levels of urinary pesticide metabolites and NB function. The increasing number of reports of NB performance deficits in workers with long-term exposure to pesticides is significant and points to the need for assurance that farmworkers receive mandated pesticide safety training and that occupational biomonitoring extend beyond those individuals who handle and apply pesticides. Finally, improved worker surveillance is needed to allow estimation of the extent of pesticide exposure among a workforce that moves frequently to meet the employment needs of multiple agricultural operations.

## Figures and Tables

**Table 1:** Organophosphate pesticides detected (μg/g) in farmworker housing in Hood River, Oregon, 1999 (*n* = 26).

	Diazinon	Methyl parathion	Chlorpyrifos	Malathion	Phosmet	AZM	Combined total^a^
No. detected (%)	20 (77)	16 (62)	24 (92)	21 (81)	25 (96)	18 (69)	
LOD	0.01	0.01	0.01	0.01	0.01	0.10	
Minimum	0.01	0.01	0.01	0.05	0.16	0.30	0.57
Mean ± SD	0.31 ± 0.23	0.38 ± 0.60	0.20 ± 0.24	0.38 ± 0.40	5.2 ± 4.1	5.9 ± 4.5	10 ± 6.5
Median	0.31	0.06	0.13	0.18	4.4	5.3	9.4
Maximum	0.72	1.9	1.2	1.4	22	16	26

a Sum of six organophosphate pesticide residues; nondetects replaced by half the LOD before summation.

**Table 2:** Urinary metabolite levels (μmol/L) in farmworkers at T1 and T2.

		Percent detect^a^		Mean ± SD^b^		Median^b^	
	LOD	T1 (*n* = 93)	T2 (*n* = 79)	T1 (*n* = 84)	T2 (*n* = 68)	T1 (*n* = 84)	T2 (*n* = 68)
DMTP	0.015	97	100	0.63 ± 0.79	0.67 ± 0.67	0.34	0.35
DMDTP	0.010	74	95	0.34 ± 0.69	0.54 ± 0.88	0.09	0.12
DETP	0.0095	34	33	0.04 ± 0.12	0.02 ± 0.03	0.00	0.00
DMTP + DMDTP^c^	—	—	—	0.97 ± 1.40	1.21 ± 1.46	0.43	0.48

—, not defined for combined quantities. Nondetects were replaced by one-half the LOD before computing summary statistics.

a Complete sample.

b Valid urine samples only.

c DMTP + DMDTP for T1 and T2 combined with at least one valid urine sample (*n* = 88): mean, 1.01 ± 1.08; median, 0.56.

**Table 3:** Partial correlations between NB performance and levels of combined thiomethyl metabolites adjusted for age, sex, and education.

Test	Partial correlation	One-sided *p*-value
Digit span forward^a^	0.122	0.861
Digit span backward^a^	0.144	0.871
Progressive ratio^a^	−0.149^b^	0.088
Reaction time^a^	0.155^b^	0.080
Selective attention trials	−0.120^b^	0.139
Selective attention ISI^a^	0.088^b^	0.214
Selective attention latency	0.251^b^	0.011
Serial digit learning^a^	0.063	0.711
Symbol-digit latency^a^	0.281^b^	0.005
Finger tapping, preferred hand^a^	−0.252^b^	0.012
Finger tapping, nonpreferred hand^a^	−0.132^b^	0.116
Finger tapping, alternating hand^a^	−0.208^b^	0.029
Continuous performance		
% Hits^a^	0.055	0.685
% Correct rejects	0.043	0.647
Hit latency	0.195^b^	0.042
False alarm latency	0.160^b^	0.092
Summary index	−0.184^b^	0.047

ISI, interstimulus interval.

a Test is a component of the summary index.

b Higher levels of metabolites associated with poorer performance.

**Table 4:** Mean score ± SE for 16 NB tests: adjusted means corresponding to a 25-year-old individual with 6 years of education in his or her country of origin.

Test	AG	Non-AG	One-sided *p*-value
Digit span forward^a^	4.12 ± 0.17	4.37 ± 0.19	0.10^b^
Digit span backward^a^	3.86 ± 0.19	4.53 ± 0.21	< 0.01^b^
Progressive ratio^a^	600.40 ± 14.53	600.22 ± 16.44	0.50
Reaction time^a^	340.95 ± 10.50	327.77 ± 11.89	0.13^b^
Selective attention trials	450.27 ± 10.03	456.16 ± 11.48	0.31^b^
Selective attention ISI^a^	397.85 ± 13.45	386.19 ± 15.40	0.23^b^
Selective attention latency	323.00 ± 6.64	315.15 ± 7.60	0.15^b^
Serial digit learning^a^			
Male	11.36 ± 1.31	8.36 ± 1.57	0.93
Female	9.33 ± 1.09	11.56 ± 1.66	0.13^b^
Symbol-digit^a^	3034.58 ± 113.74	2973.38 ± 158.38	0.38^b^
Finger tapping, preferred hand^a^			
Male	99.80 ± 2.69	96.88 ± 3.39	0.75
Female	81.68 ± 2.31	90.41 ± 3.60	0.02^b^
Finger tapping, nonpreferred hand^a^	89.22 ± 2.51	90.75 ± 2.84	0.30^b^
Finger tapping, alternating hand^a^	52.25 ± 3.00	46.72 ± 3.42	0.95
Continuous performance			
% Hits^a^	0.90 ± 0.02	0.88 ± 0.02	0.84
% Correct rejects	0.95 ± 0.01	0.97 ± 0.01	0.26^b^
Hit latency	407.82 ± 10.38	396.55 ± 11.63	0.17^b^
False alarm latency	483.36 ± 21.40	494.16 ± 24.88	0.67
Summary index			
Male	1.01 ± 0.32	0.18 (0.38)	0.95
Female	−1.00 ± 0.25	−0.04 (0.39)	0.02^b^

ISI, interstimulus interval. The one-sided *p*-value tests whether performance within the AG group is lower than within the non-AG group.

a Test is a component of summary index.

b Non-AG performed better than AG.

**Table 5:** β-Coefficients from significant interactions in a regression model used to compare NB performance between AG and non-AG groups.

Test (interaction)	β(SE)	*p*-Value
Serial digit learning (AG × age)		
Non-AG	0.26 (0.17)	0.13
AG	−0.16 (0.12)	0.20
Symbol-digit latency (AG × education)		
Non-AG	−90.87 (40.17)	0.03
AG	−197.52 (33.12)	< 0.01

For each NB test, the coefficient shows the change in average performance for each additional year of age or education.
